# Converging Proton Minibeams with Magnetic Fields for Optimized Radiation Therapy: A Proof of Concept

**DOI:** 10.3390/cancers14010026

**Published:** 2021-12-22

**Authors:** Marco Cavallone, Yolanda Prezado, Ludovic De Marzi

**Affiliations:** 1 Centre de Protonthérapie d’Orsay, Department of Radiation Oncology, Institut Curie, Campus Universitaire, PSL Research University, 91898 Orsay, France; 2 Institut Curie, Université PSL, CNRS UMR3347, Inserm U1021, Signalisation Radiobiologie et Cancer, 91400 Orsay, France; Yolanda.prezado@curie.fr; 3 Institut Curie, Campus Universitaire, PSL Research University, University Paris Saclay, INSERM LITO, 91898 Orsay, France

**Keywords:** proton minibeam radiation therapy, spatial fractionation, magnetic fields, Monte Carlo simulations

## Abstract

**Simple Summary:**

Healthy tissue tolerance to radiation is one of the main limitations in radiotherapy treatment. To broaden the therapeutic index, innovative approaches using non-conventional spatial and temporal beam structure are currently being investigated. Among them, proton minibeam radiation therapy is a promising solution that has already shown a remarkable increase in healthy tissue tolerance in various preclinical models. The purpose of this study is to propose potential strategies to further optimize proton minibeam spatial modulation with the use of magnetic fields. By generating a converging minibeam pattern with dipole magnetic fields, we show that spatial modulation can be improved at shallow depth for the same dose distribution at the tumor location. This indicates that proton minibeam radiation therapy could be efficiently combined with magnetic fields to further increase healthy tissue tolerance.

**Abstract:**

Proton MiniBeam Radiation Therapy (pMBRT) is a novel strategy that combines the benefits of minibeam radiation therapy with the more precise ballistics of protons to further optimize the dose distribution and reduce radiation side effects. The aim of this study is to investigate possible strategies to couple pMBRT with dipole magnetic fields to generate a converging minibeam pattern and increase the center-to-center distance between minibeams. Magnetic field optimization was performed so as to obtain the same transverse dose profile at the Bragg peak position as in a reference configuration with no magnetic field. Monte Carlo simulations reproducing realistic pencil beam scanning settings were used to compute the dose in a water phantom. We analyzed different minibeam generation techniques, such as the use of a static multislit collimator or a dynamic aperture, and different magnetic field positions, i.e., before or within the water phantom. The best results were obtained using a dynamic aperture coupled with a magnetic field within the water phantom. For a center-to-center distance increase from 4 mm to 6 mm, we obtained an increase of peak-to-valley dose ratio and decrease of valley dose above 50%. The results indicate that magnetic fields can be effectively used to improve the spatial modulation at shallow depth for enhanced healthy tissue sparing.

## 1. Introduction

Cancer treatment is a complex process involving many approaches, from physical removal (surgery), chemotherapy to immunotherapy or radiation therapy. Accelerated photon or electron beams are typically used during conventional radiation therapy (RT). In the last few years, clinical proton therapy (PT) has been rapidly growing, given the much-improved precision in tumor targeting and sparing of normal tissue allowed by the proton physical properties [1]. There is now increasing evidence of the influence of not only the dose, but also the dose-rate and spatial modulation of the dose on tissue response [2,3].

The concept of spatially fractionated radiation therapy (SFRT) was first introduced at the beginning of the 20th century and subsequently re-introduced in the 1970s with Co-60 machines and in the 1990s with LINACs, under the name of GRID therapy (centimeter scale pencil beams) [4,5] or microbeam radiation therapy (with extremely narrow beams of 25–100 μm) [6]. Such techniques take advantage of inhomogeneous dose profiles characterized by peaks and valleys to reduce radiation side effects. It has been demonstrated that high Peak-to-Valley Dose Ratio (PVDR) together with a low valley dose are related to better healthy tissue sparing [7]. Minibeam radiation therapy (MBRT), which employs sub-millimetric beam widths (400–1000 μm), has proven to be a good compromise between the large beams of GRID therapy and the micrometric ones of MRT, because it is easier to fulfil dosimetry and positioning requirements as well as the possibility to use higher energies than in MRT [2].

The use of charged particles such as protons or very high-energy electrons in SFRT was recently proposed, as they combine several potential advantages such as a precise ballistics, a reduction of the integral dose, and multiple Coulomb scattering of charged particles that could enable treatment of deep seated tumors with a homogeneous dose distribution, whereas normal tissues at shallow depths still benefit from spatial fractionation of the dose [8,9].

Proton MBRT (pMBRT) has been recently implemented at a clinical facility using high energy beams (≥100 MeV) with passive scattering or scanning techniques [10,11]. The experiments reported so far have shown a remarkable increase in normal tissue sparing, both for the skin and brain [12,13,14] and equivalent or superior tumor control than standard PT in preclinical models [15,16,17]. An initial theoretical investigation of the clinical potential of this technique was also conducted using a dedicated dose engine, which was developed and used to evaluate clinically relevant patients’ treatment plans [18].

The recent clinical introduction of MRI-guided radiotherapy and the integration of MRI within radiotherapy treatment machines have motivated research on potential impact of the magnetic fields on charged particle transport and dose distortions, which need to be taken into account for dose measurement, calculation, optimization, and delivery [19]. The presence of a strong magnetic field can influence the dose distribution because of the deflection of charged primary and secondary particles as well as the response of detectors used in radiation dosimetry, thus decreasing their measurement accuracy [20,21,22,23].

Finite element (numerically solving the relativistic Lorentz equations to simulate and export realistic magnetic fields maps) and Monte Carlo (radiation transport) methods can be combined and used to accurately simulate the beam transport within the treatment volume. Fast optimized analytical methods to quantify the beam deflections in the presence of magnetic fields have been proposed to predict the trajectory of mono-energetic proton beams for the purpose of MRI-PT [20,24]. Monte Carlo (MC) beam models for dose calculation with static magnetic fields were also benchmarked and validated against experimental data [21,23]. The clinical relevance of the magnetic field-induced dose disturbances in MRI-PT up to 1.5 T was finally investigated for both passive and active PT and various anatomical sites, and it was demonstrated that they could be compensated with the implementation of proper corrections to plan parameters such as small beam isocenter shifts or gantry angle rotation [25,26].

On the other hand, magnetic fields can be used to intentionally deflect or focus particle beams around the treatment isocenter: this approach has been proposed by [27], who demonstrated the feasibility of converging 160 MeV VHEE beams using external magnetic fields and two electromagnetic quadrupole triplets, or by [28,29], who proposed a new technique for generating a proton minibeam through magnetic focusing in an optimized nozzle design.

In this study, we investigate the feasibility of coupling magnetic fields with intensity-modulated proton planar minibeam therapy (pMBRT) to further optimize the characteristics of current proton minibeam delivery systems. Notably, we studied the possibility of using dipole fields to converge minibeams at the Bragg peak location and obtain higher PVDR at shallow depth with respect to classical pMBRT. The impact of the magnetic field on the beam delivery is, therefore, evaluated and adaptation strategies of PBS plan delivery and pMBRT planning are presented. Analytical models were used to determine proton trajectories in magnetic fields and optimize their intensity, while Monte Carlo simulations were performed to obtain the dose distribution in a water phantom.

## 2. Materials and Methods

### 2.1. Monte Carlo Simulations

The MC simulation code TOPAS (TOolkit for PArticle Simulation v3.5 based on Geant4.10.06.p1, [30]) was parameterized in this study to perform simulations of a real minibeam radiation therapy set-up with a proton pencil beam scanning system. In particular, all elements composing the nozzle were taken into account to simulate the full treatment head in beam scanning mode, as described in [31]. Pencil beams are modeled using their energy, energy spread, spot transverse dimension, divergence, and correlation at the vacuum window. Customized spot grids at the isocenter are obtained by setting the scanning magnet field as a function of the spot energy. Multislit divergent collimators were simulated according to the methodology described by [11] in order to produce planar minibeams. The TOPAS simulations were run with a physics list composed of seven modules: “tsem-standard_opt3”, “g4h-phy_QGSP_BIC_HP”, “g4decay”, “g4ion-binarycascade”, “g4h-elastic HP”, “g4stopping”, and “g4radioactivedecay”. For beam steering, we adopted the method described by [32] and used uniform magnetic fields within the magnets (with the “classical 4th order Runge-Kutta” stepping algorithm). Cuts for all particle productions were set to 0.01 mm. In all simulations, the parameters for the minimum and maximum EM range were set to 100 eV and 230 MeV, the number of bins per decade for stopping power and lambda bins were set to 100. A total of 1 × 10^9^ proton histories were simulated for each setup to obtain a level of relative statistical uncertainty of less than 1% at each voxel throughout the distribution. The dose is scored in a water phantom of 10 × 10 × 30 cm^3^ with a pixel dimension of 0.1 × 2 × 1 mm^3^.

### 2.2. Simulation Geometry and Configurations

**Figure 1:**
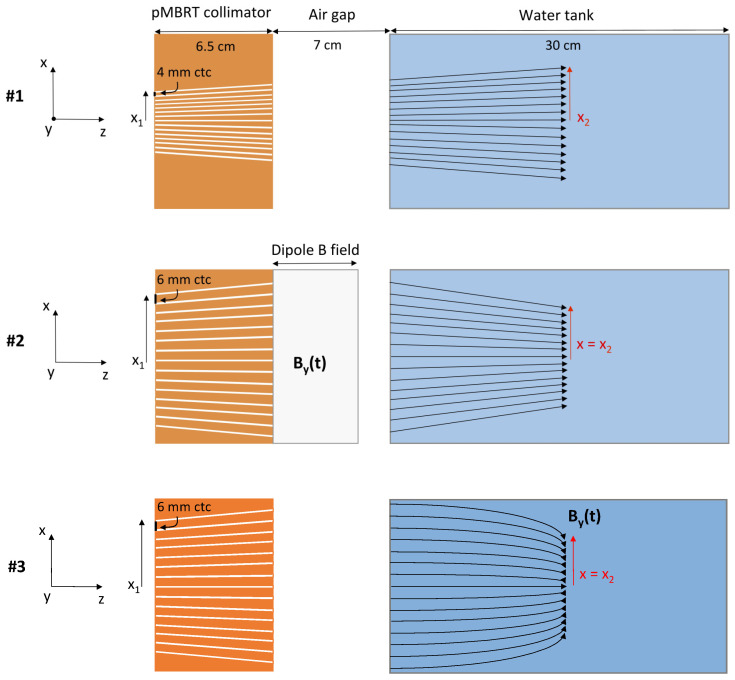
Schematic drawing of the configurations of magnetic field and collimator used in the study.

Different configurations of the last part of the beamline were simulated to investigate the possibility of using magnetic fields placed after the pMBRT collimator to create a converging minibeam pattern, and allow the use of a larger center-to-center distance for the same dose distribution at the Bragg peak location. Notably, we simulated four setups with different configurations of magnetic field and pMBRT collimator. We compared the results in terms of dose distribution in the water phantom with a classic pMBRT configuration without a magnetic field. Figure 1 shows a schematic drawing of the configurations used for the comparison.

In the reference *Configuration #1*, planar minibeams are produced with a 6.5 cm thick multislit pMBRT collimator containing 15 slits of 0.4 × 45 mm^2^ with a center-to-center (ctc) distance of 4 mm and with a slit tilt increasing linearly with the off-axis distance (0.025 degree per millimeter) to fit the beam divergence. In *Configuration #2*, a collimator with the same slit dimension, but with a larger ctc distance of 6 mm, is coupled with a 5-cm thick dipole placed after it to deviate the minibeams and ensure the same transverse dose distribution at the Bragg peak. The magnetic field in the dipole is uniform in space and directed towards the *y* direction. To converge the minibeams, the field intensity is increased with the off-axis distance of the irradiated slit (*x1* in Figure 1) by correlating the dipole field value with the pencil beam spot *x* coordinate in TOPAS simulations. The minibeams are, thus, deflected towards the central *z*-axis with an angle at the dipole exit that increases with their off-axis distance. In *Configuration #3*, we used the same collimator as in *Configuration #2*, but we deflected the minibeams by applying a homogeneous magnetic field to the water phantom, in the same manner as with an MRI-guided treatment. As in *Configuration #2*, the magnetic field is directed in the *y* direction and its intensity increases with the *x*-position of the pencil beam spots.

In all configurations, the entrance surface of the water phantom was placed at 7 cm from the collimator rear surface and a PBS grid aligned with the slit position was used. A total of 31 × 17 spots were used, with a vertical spacing of 3 mm and a horizontal spacing equal to the slit ctc distance (i.e., 4 mm in *Configuration #1* and 6 mm in *Configuration #2* and *#3*). In addition, we investigated two variants of *Configurations #2* and *#3* in which minibeams are produced with a single scanning dynamic aperture (see [33] for more details on the concept), whose off-axis *x* coordinate and tilt are correlated with the pencil beam spot *x* coordinate so as to generate the same minibeam grid as in *Configurations #2* and *#3*. With such an approach, a given pencil beam impinging on the collimator irradiates only the dynamic slit aligned with it, whereas with a static multislit, the collimator pencil beams (that have a transverse dimension of few millimeters) also irradiate nearby slits. In other words, the entire proton flux of a given minibeam produced with a dynamic slit comes exclusively from the pencil beam spots with the associated *x* coordinate. We refer to these configurations as *Configuration #2′* and *#3′* in the rest of the manuscript.

For each of the aforementioned configurations, we first investigated the effect of magnetic fields with three monoenergetic beams of 100, 150, and 200 MeV, and then we applied these approaches to a 3 cm large spread-out Bragg peak (SOBP) at a depth of 15.7–18.7 cm (relevant for clinical studies), obtained using five energy layers from 150 MeV to 162 MeV.

### 2.3. Magnetic Field Optimization

The magnetic field intensity in the dipole and in the water tank has to be correlated to the spot off-axis *x* position and is then optimized with the aim of obtaining the same transverse dose distribution at the Bragg peak compared to the reference *Configuration #1.* To do so, we first computed the off-axis *x* coordinates at the Bragg peak in *Configuration #1* for each minibeam and each energy, which depends on the range and on the slit divergence, according to:

(1) $$x_{2}=x_{1}+\left[\Delta z_{coll}+\Delta z_{air}+R\left(E\right)\right]\cdot \tan\theta \left(x_{1}\right),$$

where θ is the slit tilt and $R,\;\Delta z_{coll}$, and $\Delta z_{air}$ are the proton range, the collimator thickness, and the air gap, respectively. The value of the magnetic field needed to deflect the minibeams according to their off-axis distance is then calculated in order to have the same $x_{2}$ in all configurations. For *Configuration #2* and *#2′*, the minibeam trajectory inside the dipole as a function of the magnetic field is calculated with the *Larmour* formula r=mv/qB, where m is the proton mass, v is the proton velocity, q is the proton charge, and B is the magnetic field intensity. Knowing the radius of protons trajectory inside the dipole, the angle between the proton velocity and the beamline axis (*z*-axis in Figure 1) at the dipole exit is given by:

(2) $$\phi =\theta -\sin^{-1}\left(\Delta z_{dip}/r\right),$$

where $\Delta z_{dip}$ is the dipole thickness. The off-axis *x* coordinate at the Bragg peak $x_{2}$ can be computed using the linear equation for a straight drift:

(3) $$x_{2}=x_{de}+R\left(E\right)\cdot \tan\phi ,$$

where $x_{de}$ is the off-axis *x* coordinate at the dipole exit. Since $r\gg \Delta z_{dip}$, we assumed $x_{de}=x_{1}$.

For *Configuration #3* and *#3′*, the *x* coordinate at the Bragg peak for a given magnetic field is computed using the analytical approach proposed by [24] for a proton slowing down in a magnetic field (Equation (17) in reference [24]).

Magnetic field optimization was performed for the three beam energies used for the monoenergetic simulations as well as for the five energy layers composing the SOBP.

### 2.4. SOBP Optimization

Besides monoenergetic beams, a 3 cm wide SOBP composed of five energy layers was used to investigate these approaches with a clinically relevant scenario. An initial set of five energies between 150 MeV and 166 MeV with energy steps of 4 MeV was chosen, which corresponds to five individual Bragg peaks at a depth between 157 mm and 187 mm with a distal spacing between peaks of about 7.5 mm. We assumed as constant the weights $w_{i}$ of each pencil beam composing a given energy layer and we used a Genetic Algorithm (GA) approach to optimize the weights associated to the energy layers so as to form a homogeneous SOBP in depth. GAs are heuristic optimization processes inspired by Darwin’s natural evolution theory that are used to find optimal or near-optimal solutions to optimization problems. In these algorithms, the fittest elements (chromosomes) of a population of possible solutions are selected and combined randomly to give rise to a new child population, through an iterative process. Selection of the fittest chromosomes to be promoted and recombined is guided by a fitness function that contains the parameters to be optimized and gives a fitness score to each chromosome. A detailed description of GAs can be found in [34].

For a given configuration, Monte Carlo simulations (TOPAS) were run separately for each energy layer to obtain a library of 3D dose maps in water as a function of the energy. The weights $w_{i}$ were optimized so as to maximize the homogeneity of the depth-dose within the SOBP. To this end, the maximum dose error within the SOBP of the central minibeam peak dose was used as fitness function *f* in the GA, according to:

(4) $$f={\left|\max\left(D_{p}\right)-\min\left(D_{p}\right)\right|}_{SOBP},$$

where the peak-dose $D_{p}$ is given by the weighted sum of the dose delivered by each energy layer $D_{p}^{i}$:

(5) $$D_{p}\left(z\right)=\sum_{i=1}^{5}w_{i}\cdot \;D_{p}^{i}\left(z\right),$$

Optimization was performed for each configuration separately, in order to verify whether the optimal combination of weights is affected by the different minibeam patterns.

## 3. Results

### 3.1. Monoenergetic Beams

Table 1 reports the values of the magnetic field intensity required in the two configurations to deflect the minibeams as a function of the slit off-axis distance for the three energetic components.

In both configurations, the required field intensity increases with the slit off-axis distance and decreases with the energy, because of the lower deflection required for protons that have a larger range in water. We found maximum values of 3.48 T and 6.18 T to deflect the more external minibeams in *Configuration #2* and *Configuration #3*, respectively. Such values depend on the specific set-up, as will be discussed in Section 4, and therefore, the values presented in this study are intended for these specific configurations.

**Figure 2:**
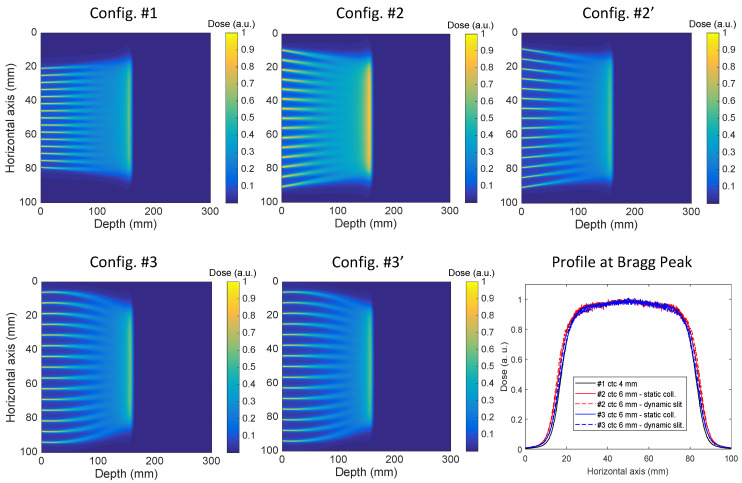
Dose maps for a 150 MeV beam in the five analyzed configurations and the corresponding transverse profiles at the Bragg peak (bottom-right figure).

As an example of the effect of the magnetic field on minibeams pattern, the 2D dose distribution in water for a 150 MeV beam and for all investigated configurations is shown in Figure 2, together with a comparison of the transverse profiles obtained at the Bragg peak.

**Figure 3:**
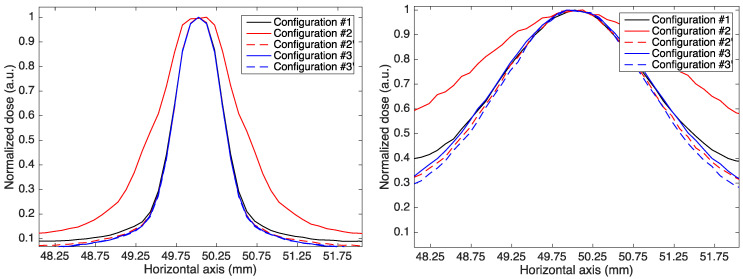
Transverse profile of the central minibeam for 150 MeV protons at the entrance of the water phantom and at a 7 cm depth.

It can be observed that the minibeams in *Configuration #2* (static multislit collimator) are larger than those in *Configuration #1* (no magnetic field) and *Configuration #2′* (dynamic aperture), indicating that the use of magnetic fields combined with a static multislit collimator leads to minibeam broadening. This is shown in Figure 3, containing the transverse profile of the central minibeam at the entrance of the water phantom and at 7 cm in depth (half of the range for 150 MeV protons) in all configurations.

The broadening effect is caused by the irradiation of a given slit by PBS spots that are not aligned with it. As a consequence, a percentage of each minibeam proton flux comes from PBS spots centered on nearby slits and is, therefore, deflected with a non-optimized magnetic field. The broadening effect is in fact eliminated when a dynamic slit is used (*Configurations #2′* and *#3′*) as the entire minibeam proton flux is generated only by PBS spots with the corresponding off-axis coordinate and is, therefore, deflected by an optimized magnetic field. When using a magnetic field inside the water phantom (*Configurations #3* and *#3′*), the difference between the use of a static collimator and a dynamic slit is less pronounced and becomes visible only after few centimeters of water thickness (Figure 3, right picture). This is because magnetic fields affect proton trajectories only after they enter the water phantom and beam widening is largely due to scattering.

The transverse dose profiles at the Bragg peak in the five configurations (bottom-right image in Figure 2) show that we managed to achieve a comparable dose distribution with respect to the reference condition with no magnetic field, with a discrepancy in the flat region within the 95% isodose of 2%. This result confirms the robustness of the approach used for magnetic field optimization.

**Figure 4:**
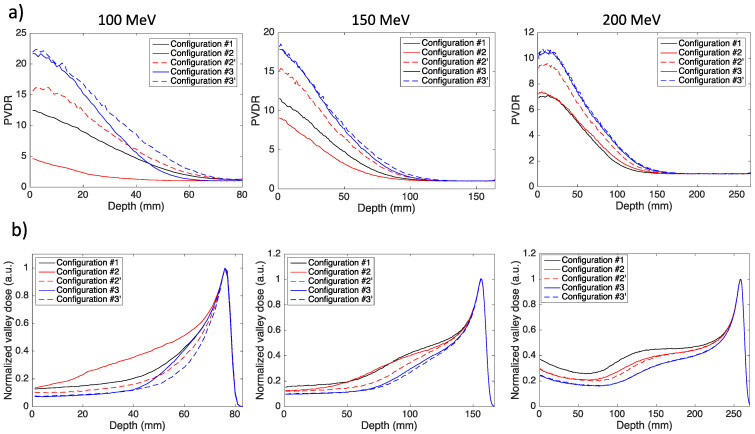
(**a**) Peak-to-Valley Dose Ratio (PVDR) as a function of depth and (**b**) depth dose of the first central valley in the five analyzed configurations for three different proton energies.

The advantage of using magnetic fields and a converging minibeam pattern can be assessed by comparing the curves of the PVDR and valley dose in depth with those of the reference *Configuration #1* without magnetic fields. Figure 4 shows the PVDR and valley dose curves in depth for the three beam energies used in the study and the five analyzed configurations. The PVDR was computed as the ratio between the peak dose on the central minibeam axis and the minimum dose in the first central valley region at the same depth.

With respect to the reference configuration, the use of a dipole and a static collimator (*Configuration #2*) degrades the PVDR, since the detrimental minibeam broadening effect is dominant with respect to the benefits of a larger ctc distance. This is evident in the PVDR curves of 100 MeV and 150 MeV. The valley dose is indeed higher than that of *Configuration #1* for the 100 MeV beam (Figure 4b, left graph, red line) and becomes comparable at 150 MeV (Figure 4b, central graph, red and black line). For higher energies, the valley dose is reduced but the PVDR remains comparable to that of *Configuration #1*, because the peak dose is also reduced. This configuration, therefore, only allows a decrease of the valley dose for energies above 150 MeV. On the contrary, the use of a dynamic slit and a dipole (*Configuration #2′*) allows an increase of the PVDR between 25% (100 MeV) and 32% (200 MeV) at the phantom entrance and between 25% and 40% at a depth equal to half the proton range, and an equivalent reduction of the valley dose. When using a magnetic field in the water phantom, both the static collimator and dynamic slit configurations provide a higher PVDR and lower valley doses compared to the reference configuration, with the second case being more favorable because of the absence of a broadening effect. The advantage of using a dynamic slit is obvious for the lower energy beam of 100 MeV, in which the PVDR curve of *Configuration #3* lies well below the curve of Configuration #3′ and decreases even below that of *Configuration #1* after a water thickness of 45 cm. An increase of PVDR and a decrease of the valley dose above 50% was obtained in *Configuration #3′* at the water phantom entrance and at half the proton range depth for all energies. Table 2 reports the PVDR values at different depths for the reference *Configuration #1* and the two configurations employing a dynamic slit.

### 3.2. Spread-Out Bragg Peak

**Figure 5:**
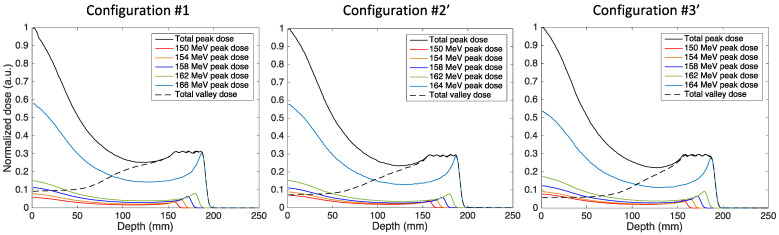
Optimized spread-out Bragg peak in the three configurations. The figure shows the contributions of the individual Bragg peaks (colored continuous lines) to the total peak dose (black continuous line) on the central minibeam axis as well as the valley dose on the first central valley (dashed black line).

Since the best results with monoenergetic beams were obtained using a dynamic aperture, we studied the creation of an SOBP only in *Configuration #1*, *Configuration #2′,* and *#3′*. Figure 5 shows the depth-dose profiles of the optimized SOBPs in these three configurations.

The graphs show the total depth dose on the central minibeam axis and on the first central valley as well as the contribution of the individual Bragg peaks on the peak dose. In all cases, a maximum dose error within the SOBP below 3% was achieved with the genetic optimization. As mentioned above, the optimization of the weights $w_{i}$ was performed for each configuration, since the different initial ctc distance and minibeam pattern during the slowing down can affect the spectral tailoring of the beam. The optimized weights in the three configurations are reported in Table 3.

As expected, the optimization does not lead to the same spectral tailoring: the weights of the shorter range layers, normalized to the last layer weight, are higher in *Configuration #2′* and *#3′* compared to *Configuration #1*. This can be explained by considering that in *Configuration #2′* and *#3′*, the individual Bragg peaks with a higher range have a larger transverse profile at the beginning of the SOBP compared to the configuration without magnetic field, since minibeams have a converging pattern; in addition, their trajectory is optimized to obtain the same transverse profile at the corresponding individual Bragg peak depth. Therefore, the dose contribution of the higher energetic components at the SOBP entrance is lower with a converging minibeam pattern than with straight minibeams and must be compensated by a higher contribution of the lower energetic components to achieve a flat SOBP in depth. This suggests that, in future clinical applications, optimization of the spot weights must account for the different minibeam pattern in the presence of magnetic fields, either by adapting current treatment planning systems or by post-processing correction of the optimization performed without a magnetic field.

**Figure 6:**
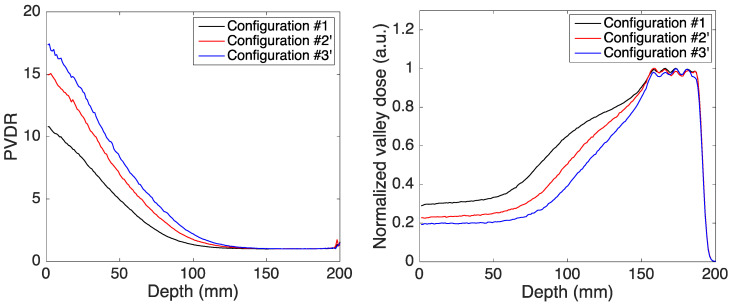
Peak-to-valley dose ratio as a function of depth (**left image**) and depth-dose of the first central valley (**right image**) in the three configurations.

The PVDR and valley dose curves obtained in the three configurations are shown in Figure 6.

As in the case of monoenergetic beams, the use of magnetic fields allows for higher PVDR (lower valley dose) at shallow depths for a comparable PVDR (valley dose) at the SOBP location. A decrease of the valley dose of 30% and 50% was obtained with the dipole field placed after the pMBRT collimator (*Configuration #2′*) and within the water phantom (*Configuration #3′*), respectively. More importantly, the use of a converging minibeam pattern would allow spatial fractionation in healthy tissues close to the tumor. In the scenario discussed, a healthy tissue placed at a depth of 10 cm would benefit from a fractionated dose pattern with a PVDR of 2.3 (blue curve in Figure 6), whereas it would receive a nearly homogeneous dose (PVDR of 1.3) in the classical configuration with no magnetic field.

## 4. Discussion

In this study, we examined for the first time the use of dipole magnetic fields to converge planar minibeams in depth with the goal of increasing the initial ctc distance between minibeams. This method was shown to improve the spatial modulation on healthy tissues for the same transverse dose homogeneity at the Bragg peak location.

In all configurations (except *Configuration #2*), the increase in PVDR is related with an equivalent drop of the valley dose due to the larger separation between minibeams. This is a significant result because the valley dose and ctc distance seem to have a significant impact on healthy tissue tolerance in SFRT techniques [35,36,37]. Among the configurations investigated, the use of a magnetic field inside the water phantom, mimicking an MRI-guided machine, is the most effective approach to optimize the spatial modulation. Since protons are increasingly deflected as they slow down in matter, the distance between minibeams before the Bragg peak position is larger in comparison to the setup in which protons are deflected with a dipole magnet placed before the water phantom. It is worth mentioning, however, that this comparison is conducted with the same ctc distance between collimator slits. Minibeams deflected with the dipole magnet, thus, have a shorter ctc distance at the water tank entrance than when they are deflected after entering the water phantom. If the comparison were conducted with the same ctc distance at the water tank entrance (i.e., by adapting the slit ctc distance in the two configurations), the PVDR of *Configuration #2′* at shallow depth would be comparable to that of *Configuration #3′* and the difference between the two curves in depth would be reduced.

We also investigated the combination of both a static multislit collimator and a dynamic single scanning aperture with magnetic fields. The use of a dynamic aperture provides the best results in terms of the increase of PVDR and reduction of valley dose because the minibeam broadening (due to the deflection of a fraction of each minibeam flux with a non-optimized magnetic field intensity) is avoided. The generation of planar minibeam arrays with a dynamic aperture has been recently investigated at our facility and more details can be found in [33]. Furthermore, a scanning aperture would ease a practical implementation of the approaches presented: it could be irradiated with a field consisting of a few adjacent spots at each scanning aperture position so as to reduce the sensitivity to misalignment between the PBS spots and the slit. Conversely, a successful implementation of these techniques with a multislit static collimator relies on the ability to align the PBS spots with the slits, which is more challenging to achieve in real practice and more sensitive to misalignments. Moreover, our study was carried out considering the pencil beam dimensions of an existing clinical machine, in the presence of a static collimator. Further studies should also investigate these approaches in the case of irradiation with magnetically focused minibeams, which could be even smaller and obtained without a mechanical collimator [28,29]. It is worth observing that minibeam width is not significantly affected by dipole magnetic fields, except when a dipole is coupled with a static multislit collimator (*Configuration #2*).

Another factor to consider for a practical implementation of the approaches presented is the maximum value of magnetic field required to deflect minibeams. The magnetic field intensity must be increased with the off-axis distance of the minibeam; therefore, the larger the increase in ctc distance with respect to the reference configuration (with no magnetic field), the stronger the maximum magnetic field required to achieve the same transverse Bragg peak dimension and homogeneity. Likewise, for a given increase in ctc distance, the larger the transverse area of the target volume, the higher the number of minibeam arrays required and consequently the maximum magnetic field. Moreover, the magnetic field intensity decreases with the beam energy. This is due to the lower deflection angle required for protons having a larger range in depth. Therefore, constraints on the maximum magnetic field would translate into limitations on the minimum beam energy, the number of slit and the transverse dimension of the target area. For the configurations investigated in this study, we found a maximum intensity required for the 100 MeV beam of 3.48 T in the case of a dipole placed after the pMBRT collimator and of 6.18 T if the magnetic field is applied to the water phantom in an MRI-PT guided-like scenario. We should stress that these values also depend on the distance travelled by particles in the magnetic field region. As a result, while the magnetic field extension in the MRI-PT scenario is determined by the proton range, the magnetic field intensity in the dipole placed before the water phantom can be reduced by increasing the dipole thickness. Currently, commercially available MRI-based systems exist only for LINAC, and typical magnetic fields are below 1.5 T [19,38,39]. Therefore, limitations on the magnetic field in MRI-PT machines must be considered for a practical implementation of this configuration at energies below 150 MeV or for a transverse dimension of the treatment volume larger than few centimeters.

For this proof of concept, we assumed a homogeneous magnetic field. This is not a difficult condition to meet with a dipole placed after the pMBRT collimator, considering that the homogeneous region can be moved to follow the slit during the scan. Concerning the MRI-PT scenario, a good homogeneous field has been reported for an MRI-LINAC system within a volume of 50 × 50 × 50 cm^3^ [40]. However, fringe fields outside the deflection region were not taken into account in this study. Magnetic field in fringe field regions can produce additional proton deflection not only along the desired direction but also along the perpendicular direction, since other components of the magnetic field might not be negligible in this region [40]. For a more in-depth investigation of these approaches, future studies should integrate real field maps and assess to what extent fringe fields affect the proton trajectories and the magnetic field optimization strategy.

The Bragg peak retraction in the z direction, due to the deflected trajectory, is another aspect to include in a 3D dose optimization process with such techniques. In the cases presented, the Bragg peak retraction has a negligible impact on the transverse Bragg peak optimization and was not taken into account. It is limited to a maximum of 2 mm for external minibeams, in agreement with the values found by [24,41] for comparable magnetic fields and beam energies.

## 5. Conclusions

We investigated different configurations of coupling dipole magnetic fields with planar minibeams to produce a converging pattern in depth and improve the spatial modulation at shallow depth. We showed that using a static multislit collimator coupled with a dipole magnet placed after it degrades the PVDR because of the non-optimized interaction between the magnetic field and multiple minibeams at the same time. The use of a dipole magnet is advantageous only when coupled with a dynamic scanning aperture, in which case we obtained an increase of PVDR and decrease of valley dose up to 30% at the phantom entrance for the same transverse dose homogeneity at the Bragg-peak location. The use of magnetic fields in the water phantom is less affected by the minibeam broadening effect due to non-optimized magnetic fields and provides a considerable improvement of spatial modulation at shallow depth with both a static and a dynamic collimator. In this second case, we obtained an increase of PVDR (and decrease of valley dose) above 50%. A relevant improvement of spatial modulation at shallow depth was also obtained with a more complex set-up employing five energy layers from 150 to 166 MeV, generating a 3 cm wide SOBP relevant for clinical applications. Altogether, these results show that pMBRT could be efficiently combined with magnetic fields to further improve the spatial modulation on healthy tissues, provided that a practical implementation is studied in detail.

## Figures and Tables

**Table 1:** Intensity of the magnetic field optimized for the two configurations as a function of the slit off-axis distance and the beam energy.

Magnetic Field	Slit Off-Axis Distance (mm)	100 MeV Beam	150 MeV Beam	200 MeV Beam
**Configuration #2**	6	0.5 T	0.37 T	0.279 T
	12	1 T	0.74 T	0.558 T
	18	1.5 T	1.105 T	0.837 T
	24	2 T	1.475 T	1.116 T
	30	2.5 T	1.84 T	1.395 T
	36	2.99 T	2.21 T	1.674 T
	42	3.48 T	2.575 T	1.953 T
**Configuration #3**	6	0.883 T	0.274 T	0.121 T
	12	1.766 T	0.548 T	0.243 T
	18	2.649 T	0.822 T	0.364 T
	24	3.532 T	1.096 T	0.486 T
	30	4.415 T	1.37 T	0.607 T
	36	5.298 T	1.644 T	0.728 T
	42	6.181 T	1.918 T	0.85 T

**Table 2:** PVDR as a function of depth for the three beam energies and three configurations.

	Depth in Phantom	Configuration #1	Configuration #2′	Configuration #3′
100 MeV	Phantom entrance	12.5 ± 0.1	15.6 ± 0.1	22.3 ± 0.2
	3.8 cm	5.07 ± 0.05	6.35 ± 0.06	9.18 ± 0.09
	7.5 cm (BP)	1.22 ± 0.01	1.26 ± 0.01	1.30 ± 0.01
150 MeV	Phantom entrance	11.6 ± 0.1	15.1 ± 0.1	18.2 ± 0.2
	3.8 cm	6.42 ± 0.06	8.59 ± 0.09	10.4 ± 0.1
	7.5 cm	2.20 ± 0.02	3.10 ± 0.03	4.09 ± 0.04
	12.5 cm	1.03 ± 0.01	1.07 ± 0.01	1.12 ± 0.01
200 MeV	Phantom entrance	6.98 ± 0.07	9.22 ± 0.09	10.3 ± 0.1
	3.8 cm	5.91 ± 0.06	7.77 ± 0.08	8.71 ± 0.09
	7.5 cm	3.24 ± 0.03	4.45 ± 0.04	5.21 ± 0.05
	12.5 cm	1.21 ± 0.01	1.58 ± 0.02	1.89 ± 0.02
	15 cm	1.05 ± 0.01	1.14 ± 0.01	1.27 ± 0.01

**Table 3:** Normalized weights $w_{i}$ obtained with genetic optimization of the 3 cm wide SOBP in the reference *Configuration #1* and in the two configurations with magnetic fields and a dynamic aperture.

Weights *w_i_*	150 MeV	154 MeV	158 MeV	162 MeV	166 MeV
Configuration #1	0.104	0.140	0.202	0.263	1
Configuration #2′	0.141	0.171	0.210	0.275	1
Configuration #3′	0.167	0.190	0.248	0.338	1

## Data Availability

Data are contained within the article.
